# A systematic review of the epidemiology of carbapenem-resistant Enterobacteriaceae in the United States

**DOI:** 10.1186/s13756-018-0346-9

**Published:** 2018-04-24

**Authors:** Daniel J. Livorsi, Margaret L. Chorazy, Marin L. Schweizer, Erin C. Balkenende, Amy E. Blevins, Rajeshwari Nair, Matthew H. Samore, Richard E. Nelson, Karim Khader, Eli N. Perencevich

**Affiliations:** 1 Center for Comprehensive Access Delivery & Research, Iowa City VA Health Care System, Iowa City, USA; 2 Department of Internal Medicine, Carver College of Medicine, University of Iowa, Iowa City, USA; 3 University of Iowa College of Public Health, Iowa City, USA; 4 Hardin Library for the Health Sciences, University of Iowa, Iowa City, USA; 5 Ruth Lilly Medical Library, Indiana University School of Medicine, Indianapolis, Indiana USA; 6 Veterans Affairs Salt Lake City Health Care System, Salt Lake City, Utah USA; 7 Department of Internal Medicine, University of Utah School of Medicine, Salt Lake City, Utah USA

**Keywords:** Carbapenem-resistant Enterobacteriaceae, United States, Epidemiology

## Abstract

**Background:**

Carbapenem-resistant Enterobacteriaceae (CRE) pose an urgent public health threat in the United States. An important step in planning and monitoring a national response to CRE is understanding its epidemiology and associated outcomes. We conducted a systematic literature review of studies that investigated incidence and outcomes of CRE infection in the US.

**Methods:**

We performed searches in MEDLINE via Ovid, CDSR, DARE, CENTRAL, NHS EED, Scopus, and Web of Science for articles published from 1/1/2000 to 2/1/2016 about the incidence and outcomes of CRE at US sites.

**Results:**

Five studies evaluated incidence, but many used differing definitions for cases. Across the entire US population, the reported incidence of CRE was 0.3–2.93 infections per 100,000 person-years. Infection rates were highest in long-term acute-care (LTAC) hospitals. There was insufficient data to assess trends in infection rates over time. Four studies evaluated outcomes. Mortality was higher in CRE patients in some but not all studies.

**Conclusion:**

While the incidence of CRE infections in the United States remains low on a national level, the incidence is highest in LTACs. Studies assessing outcomes in CRE-infected patients are limited in number, small in size, and have reached conflicting results. Future research should measure a variety of clinical outcomes and adequately adjust for confounders to better assess the full burden of CRE.

**Electronic supplementary material:**

The online version of this article (10.1186/s13756-018-0346-9) contains supplementary material, which is available to authorized users.

## Background

The Centers for Disease Control and Prevention (CDC) in the United States has deemed carbapenem-resistant Enterobacteriaceae (CRE) an urgent public health threat that requires immediate and aggressive action [1]. The reason for this urgency is clear: CRE infections are resistant to nearly all antibiotics and are associated with poor clinical outcomes [2].

Carbapenem-resistance in Enterobacteriaceae can result from a variety of mechanisms [3]. In the US, CRE isolates commonly produce carbapenemases; these enzymes are carried on plasmids and can be easily shared with other gram-negative bacteria. The *Klebsiella pneumoniae* carbapenemase (KPC) was first recognized in North Carolina in 1996 and has since spread around the world [4]. Other examples of carbapenemases include the New Delhi Metallo-β-lactamase (NDM), Verona Integron-encoded Metallo-β-lactamase (VIM), Oxacillinase-48-type carbapenemases (OXA-48), and imipenemase (IMP).

CRE infections are typically seen in patients with prior healthcare exposure, and medical devices are a common risk factor for CRE acquisition [5]. According to 2009–2010 data from the National Healthcare Safety Network (NHSN), 20% of hospitals reporting central line-associated bloodstream infections (CLABSIs) or catheter-associated urinary tract infections (CAUTIs) due to *Klebsiella* spp. reported at least 1 carbapenem-resistant strain [6]. In 2013, an outbreak of NDM-producing *Escherichia coli* was associated with duodenoscopes at an Illinois hospital [7].

Despite ongoing control efforts, CRE has become prevalent in several US regions, including Orange County, California and the Chicago metropolitan area [8, 9]. CRE has been endemic in the New York/New Jersey area since the early 2000s [10]. According to the 2013 CDC Antibiotic Resistance Threat Report, there are 9000 healthcare-associated CRE infections every year in the US, resulting in 600 deaths (mortality rate 6.6%) [1]. However, such a low proportion of deaths may be an underestimation due to how infections were defined.

The CDC has published toolkits on preventing the spread of CRE within and between healthcare facilities [3, 11]. While these toolkits have aided efforts at CRE prevention, a better understanding of the epidemiology and burden of CRE is critical to encourage increased investments in the study and prevention of these pathogens. To this end, we conducted a systematic review and evaluation of studies which investigated incidence and outcomes of CRE infection at US sites.

## Methods

### Search strategy

This systematic literature review was performed according to the MOOSE and PRISMA guidelines [12, 13]. An experienced health sciences librarian conducted systematic searches in MEDLINE via Ovid, Cochrane Database of Systematic Reviews via Wiley (CDSR), Database of Abstracts of Reviews of Effects via Wiley (DARE), Cochrane Central Register of Controlled Trials via Wiley (CENTRAL), NHS Economic Evaluation Database (NHS EED) via Wiley, Scopus, and Web of Science. We searched for articles published between the dates January 1, 2000 and February 1, 2016. Search terms included Medical Subject Headings (MeSH) and keywords for carbapenem resistance, Enterobacteriaceae, adverse events, incidence, prevalence, and economics. Additional file 1 includes a description of the complete search strategy. We reviewed the reference lists from each article to identify additional studies.

### Inclusion and exclusion criteria

Studies were included if they (1) were conducted in the United States; (2) evaluated incidence of CRE or outcomes attributed to CRE infections including mortality, length of stay (LOS), discharge to a long-term acute-care (LTAC) hospitals, readmission, recurrence, and costs; (3) included at least 7 study sites (incidence studies only); and (4) used either the CDC’s original or revised definition for CRE [3, 11]. In the original definition, an Enterobacteriaceae isolate qualified as CRE if it was non-susceptible to imipenem, meropenem or doripenem AND resistant to all of the following third-generation cephalosporins: ceftriaxone, cefotaxime and ceftazidime [11]. In the revised 2015 definition, an Enterobacteriaceae isolate qualified as CRE if it was resistant to imipenem, meropenem, or doripenem, (minimum inhibitory concentration [MIC] ≥ 4 mcg/mL) OR resistant to ertapenem (MIC ≥2 mcg/mL) OR had documented production of a carbapenemase [3]. In 2010, the Clinical and Laboratory Standards Institute (CLSI) lowered the breakpoints for carbapenems by a factor of 4, so in reviewing each study, we made note of which version of the CLSI breakpoints was used.

We excluded studies that (1) were less than 6 months in duration; (2) used International Classification of Diseases Clinical Modification diagnosis codes to define CRE infections; (3) used surveillance cultures from non-sterile body sites to detect CRE; (4) did not have a denominator or control group; (5) took place during an outbreak; (6) were mathematical models; (7) did not contain original data; and (8) were published in a language other than English. We excluded studies evaluating LOS or costs if they did not measure post-infection LOS or costs, or if they did not match infected cases and uninfected controls on time at risk (time from admission to infection for cases, time from admission to discharge for uninfected controls) or did not match on propensity scores.

### Data extraction and quality assessment

One investigator (MLC) reviewed the titles and abstracts of all articles for inclusion and exclusion criteria. Two of four reviewers (MLC, RN, ENP, MLS) independently abstracted data for each included study. Disagreements between reviewers were resolved by consensus.

The reviewers abstracted data on the following items: study design, population and setting, location, definition of CRE infection, incidence data, prevalence data, mortality, LOS, discharge to LTAC, readmission, recurrence, and cost. Additional data were collected regarding tests used to identify CRE infection, identification of different species that were identified as CRE, and definitions of resistance.

Risk of bias was assessed using the Newcastle-Ottawa scale for included studies [14].

## Results

**Fig. 1:**
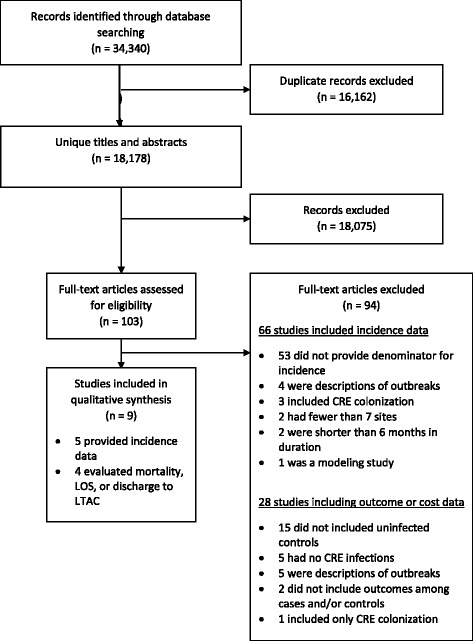
Flow diagram of search strategy. Legend: CRE, Carbapenem-resistant *Enterobacteriaceae*; LOS, length of stay; LTAC, long-term acute-care hospital

We screened 18,178 unique studies for eligibility (Fig. 1). Nine studies were eligible for inclusion, including 5 multicenter studies reporting the incidence of CRE infections [15–19] and 4 studies (2 multicenter and 2 single center) evaluating relevant outcomes [20–23]. Included studies were of moderate-to-high risk of bias according to the Newcastle-Ottawa tool (Table 1). Included studies had low risk of bias when it came to representativeness and ascertainment of CRE infected patients and controls. However, most of the included studies had high risk of bias in terms of adequacy of follow-up, cohort design and analysis, and assessment of outcomes.

**Table 1:** Risk of bias assessment of CRE studies using Newcastle Ottawa tool (Stang 2010). Receiving a star (*) represents that the study has low risk of bias and high quality in that category

	Selection				Comparability	Outcome		
Author (Year)	Representative-ness of the exposed cohort	Selection of the non-exposed cohort	Ascertainment of exposure	Demonstration that outcome of interest was not present at start of study	Comparability of cohorts on the basis of design or analysis	Assessment of outcome	Was follow-up long enough for outcomes to occur	Adequacy of follow-up of cohorts
Bogan (2014) [20]	*	*	*	*	*	*	*	_
Brennan (2014) [15]	*	*	*	*	_	_	_	_
Gasink (2009) [21]	*	*	*	*	*	*	*	_
Guh (2015) [18]	*	*	*	*	_	_	_	_
Lesho (2015) [16]	*	*	*	*	_	_	_	_
Marquez (2013) [17]	*	*	*	_	_	_	_	_
Patel (2008) [22]	*	*	*	*	*	*	*	_
Thaden (2014) [19]	*	*	*	*	_	_	_	_
Kalpoe (2012) [23]	*	*	*	*	*	*	*	*

### Incidence of CRE infections

There were 5 studies (Table 2)  that measured the incidence of CRE: 4 studies used the lower CLSI breakpoints (i.e. 2010 and later) [15–18] while one study largely used the pre-2010 breakpoints [19]. All studies included clinical cultures from sterile and non-sterile body sites, but one study limited non-sterile cultures to only urine samples [18].

**Table 2:** Studies that Evaluated CRE Incidence

First Author (Year)	Study Population	CLSI protocol	Study Period	Culture type	# of Infections	IncidenceRate
Brennan (2014) [15]	CRE Surveillance and Prevention Initiative in Michigan	M100-S22 2012	09/2012–02/2013	All cultures positive for carbapenem non-susceptible *K. pneumoniae* or *E.coli.* All cases had a positive or unknown modified Hodge-Test result. Only 1 case per patient per 30-day period.	102	Per 10,000 patient-days Overall: 1.07 Acute care: 1.01 LTAC: 2.93 East: 1.24 West: 0.52 Mid-North: 0.36
Lesho (2015) [16]	Tricare, i.e.civilian component of the US military health care system	M100-S20–2010	01/2005–12/2012	All CRE-positive cultures (*E.coli*, *Klebsiella* spp., *Enterobacter* spp.). For each year of surveillance, the first CRE-positive culture per patient was included.	368	Per 100,000 person-years All Years: 0.487 2012: 0.394 2011: 0.528 2010: 0.672 2009: 0.607 2008: 0.439 2007: 0.424 2006: 0.490 2005: 0.335
Marquez (2013) [17]	Los Angeles County	M100-S20 2010	06/2010–05/2011	All cultures positive for carbapenem-resistant *K. pneumoniae.* Only 1 isolate allowed per patient per calendar month.	675	Per 1000 patient-days Acute and LTAC: 0.46 LTAC only: 2.54 Acute only: 0.31
Thaden (2014) [19]	Duke Infection Control Outreach Network (DICON)	M100-S20 2010 (20%) sites; earlier CLSI definitions (80% sites)	01/2008–12/2012	All CRE-positive cultures. Only 1 culture was allowed per patient for the entire surveillance period.	180	Per 100,000 patient-days 2008: 0.26 2012: 1.4
Guh (2015) [18]	Multi-site Gram-negative Surveillance Initiative	M100-S22 2012	1/2012–12/2013	All CRE non-susceptible cultures (*E.coli*, *Klebsiella* spp., *Enterobacter* spp.) from a normally sterile site or urine. An incident case was defined as the first isolate from a patient per 30-day period.	599	Per 100,000 persons 2012: 2.94 2013: 2.93

*CLSI* Clinical and Laboratory Standards Institute, *CRE* carbapenem-resistant Enterobacteriaceae, *LTAC* long-term acute-care hospital

Three studies used the denominator of patient-days [15, 17, 19], but these 3 studies could not be pooled because definitions varied: 1 study included all species of Enterobacteriaceae while 2 studies used less inclusive definitions. Among these 3 studies, the incidence of CRE ranged from 0.46 CRE infections per 10,000 patient-days to 4.17 CRE infections per 10,000 patient-days. Two studies stratified by long-term acute care (LTAC) hospitals and acute care hospitals [15, 17]. One of these studies--which took place in Los Angeles County, California—found that the incidence of CRE infections in acute care hospitals was 0.31 per 10,000 patients-days and the incidence of CRE infections in LTAC hospitals was 2.54 per 10,000 patient-days [17]. The other study, which took place in Michigan, found a similar incidence of CRE infections in LTAC hospitals (2.93 per 10,000 patient-days) but the incidence of CRE infections among the acute care hospitals (1.01 per 10,000 patient-days) was higher than that of the Los Angeles County study [15].

Two studies included both inpatients and outpatients, reflected in a denominator of person-years [16, 18]. In a study of the civilian component of the US military health care system, the incidence of CRE remained steady at 0.49 infections per 100,000 person-years between 2005 and 2012; the peak incidence was 0.67 per 100,000 person years in the year 2010 [16]. A study across 7 metropolitan areas estimated an incidence of 2.94 cases per 100,000 persons in 2012 and 2.93 per 100,000 persons in 2013. Site-specific incidence rates ranged from 0.82 cases per 100,000 persons in Oregon to 4.80 cases per 100,000 persons in Maryland [18].

Carbapenemase production was assessed in 2 studies [15, 18]. One study only included isolates with a positive or unknown result on the Modified-Hodge Test (MHT), but a breakdown of MHT results was not provided [15]. In another study, 90 of 188 (47.9%) isolates across 6 sites produced a carbapenemase, all of which were KPCs [18].

### Outcomes attributable to CRE infections

Each of the 5 outcomes studies included data from 2009 or earlier (Table 3). Two studies evaluated outcomes in CRE patients compared to uninfected controls [20, 23]. In the earliest study, all patients who underwent liver transplantation at a single center were followed for 1 year after transplantation [23]. That study found that 71% of the patients infected with carbapenem-resistant *K. pneumoniae* (CRKP) died compared to 14% of the uninfected patients (log rank *p* < 0.001). Based on a multivariate Cox proportional hazards analysis, mortality at 1-year was significantly higher in patients who developed CRKP infections compared to uninfected patients (hazard ratio = 4.9, 95% CI 1.5–15.6). In the second study, patients with and without infections were matched by hospital or facility, unit or clinic, calendar year, and time at risk (i.e., from admission to culture). This study found higher odds of dying among CRE patients compared with uninfected controls, but the difference was not statistically significant (OR = 1.6; 95% CI: 0.7–3.3) [20]. The study also found that CRE infected patients had significantly higher odds of being discharged to a LTAC after being admitted from home (OR = 13.7; 95% CI: 4.3–44.4). There was no difference in LOS between CRE and uninfected patients.

**Table 3:** Studies of the Association between CRE Infection and Outcome

First Author (Year)	Study Population	CLSI protocol	Study Period	No. of Patients	Types of infections	Mortality: CRE versus control (%) HR or OR (95% CI)	Length of stay (LOS), median (IQR), days	Discharge to a LTAC after being admitted from home (%), OR (95% CI)
CRE infected patients versus uninfected controls								
Bogan (2014) [20]	Detroit Medical Center, 8 hospitals	M100-S19 2009	09/2008–08/2009	91 cases, 91 controls (matched)	All classified by NHSN definitions.^1^	All-cause in-hospital 38.3% versus 16.7% (*p* = 0.006), adjusted OR = 1.6 (0.2–14.8)^2^	CRE Infected Pts Median = 10 days (IQR: 4–23) Uninfected Pts Median = 13 days (IQR: 3–25)	OR = 15.1 (3.1–73.5)
Kalpoe (2012) [23]	Mount Sinai Hospital, New York City	Not stated	10/2005–10/2006	14 cases, 161 controls	Cultures positive for carbapenem-resistant *K.pneumoniae* from normally sterile body-sites	All-cause mortality at 1-year: 71% versus 14% (log rank *p* < 0.001) HR = 4.9 (1.5–15.6)		
Carbapenem-resistant *Enterobacteriaceae (CRE)* versus carbapenem-susceptible *Enterobacteriaceae (CSE)*								
Gasink (2009) [21]	University of Pennsylvania (2 hospitals)	KPC-production^3^	10/2006–4/2008	56 cases, 863 controls	Clinical cultures with *K.pneumoniae*^4^	All-cause in-hospital: 32.1% versus 9.9% (*p* < 0.001), adjusted OR 2.28 (1.18–4.40)		
Patel (2008) [22]	Mount Sinai Hospital, New York City	M100-S16 2006	7/2004–6/2006	99 cases, 99 controls (matched)	Cultures positive for *K. pneumoniae* from normally sterile body-sites	All-cause in-hospital: 48% versus 20%, OR 3.71 (1.97–7.01) Attributable in-hospital: OR 4.50 (2.16–9.35)		
Bogan (2014) [20]	Detroit Medical Center, 8 hospitals	M100-S19 2009	09/2008–08/2009	91 cases, 91 controls (matched)	CRE or non-ESBL CSE. All infections classified by NHSN definitions.^1^	All-cause in-hospital: 38.3% versus 16.7% (*p* = 0.004), adjusted OR 2.7 (0.8–9.4)	Among CRE Pts Median = 10 days (IQR: 4–23) Among CSE Pts Median = 7 days (IQR: 5–16)	OR = 14.5 (2.7–79.8)

*CI* confidence interval, *CRE* carbapenem-resistant Enterobacteriaceae, *CSE* carbapenem-susceptible Enterobacteriaceae, *HR* hazard ratio, *IQR* interquartile range, *KPC Klebsiella pneumoniae* carbapenemase, *LTAC* long-term acute-care facility, *NHSN* National Healthcare Safety Network, *OR* odds ratio

^1^Infectious clinical syndrome for CRE cases: 28.2% colonization, 19.7% UTI, 19.7% pneumonia, remaining syndromes not stated

^2^Excludes uninfected colonized-only CRE patients and their matched controls

^3^All cases had confirmed production of a *Klebsiella pneumoniae* carbapenemase (KPC) by either PCR for bla_KPC_ or the Modified Hodge Test

^4^Body site of positive-culture: urine 59.9%, blood 17.3%, respiratory tract 12.1%, abdomen 8.1%, other 6.6%

Three studies evaluated outcomes in patients with CRE compared to carbapenem-susceptible Enterobacteriaceae (CSE) [20–22]. Two studies only included isolates of *K. pneumoniae* while the third study included all types of CSE not producing extended-spectrum beta-lactamases (ESBLs). Two studies used multivariable analysis to adjust for important predictors of mortality; only one of these studies adjusted for severity of illness [21] while the other matched cases-and-controls on baseline factors [20]. The third study matched patients with CRE to those with CSE based on body-site of infection [22].

In both studies that exclusively evaluated *K. pneumoniae*, cases with carbapenem-resistance had significantly higher odds of all-cause in-hospital mortality (OR = 2.28 and OR = 3.71; Table 3) [21, 22]. One of these studies also found that the odds of attributable in-hospital mortality were more than 4-fold higher among CRKP-infected patients compared with carbapenem-susceptible *K. pneumoniae* infected controls [22]. In the study that included all types of Enterobacteriaceae, carbapenem-resistance was not significantly associated with either an increase in hospital mortality (adjusted OR 2.7, 95% CI 0.8–9.4) or a longer LOS. However, carbapenem-resistance was associated with being discharged to a LTAC after being admitted from home (OR = 14.5, 95% CI 2.7–79.8).

## Discussion

Estimating the incidence of CRE is an important step in designing a national public health response to this emerging pathogen [24]. Our review found that the reported incidence of CRE in the US was 0.3–2.93 infections per 100,000 person-years. The incidence of CRE is relatively higher in LTACs compared to acute-care hospitals and community settings. In 1 population-based study, nearly half of CRE isolates produced a carbapenemase. Carbapenemase-producing CRE are of the greatest epidemiologic concern, because these enzymes are typically carried on mobile genetic elements that can be shared with other bacteria [25].

Based on our findings, CRE is still relatively uncommon in the US compared to other antibiotic-resistant pathogens. For example, the estimated incidence of invasive methicillin-resistant *Staphylococcus aureus* (MRSA) infections in 2011 across the US was 25 per 100,000 persons, or at least 8 times more common than CRE [26]. In the Veterans Health Administration during 2009–2013, the overall incidence of *C. difficile* was approximately 200 infections per 100,000 patient-years, which was at least 65 times more common than CRE [27]. It is important to note that, even though the current incidence of CRE is low, CRE has been rapidly spreading across the US. Prior to 1996, carbapenemase-producing CRE was not reported in the US, but as of August 2017, this pathogen has been reported in every US state but Idaho [28].

Despite its low incidence, CRE remains a public health threat due to its limited treatment options and worse clinical outcomes [1]. Based on the studies that compared outcomes between CRE and uninfected patients, infection with CRE was associated with a higher risk of being discharged to a LTAC and a higher risk of death within the year after liver transplantation [20, 23]. Studies consistently reported an unadjusted CRE-related mortality rate that was higher than the 6.6% estimate from the CDC [1].

Surprisingly, however, patients with CRE were not always found to have an increased risk for death compared with controls. Studies that compared CRE to uninfected patients reached different conclusions on mortality, albeit using different definitions of mortality [20, 23]. Conflicting results on mortality were also seen in studies that compared CRE to CSE. Two studies found that patients with carbapenem-resistant *K. pneumoniae* had 2–3 times the odds of in-hospital death as patients with carbapenem-susceptible *K. pneumoniae*, but a similar difference in mortality was not seen in a study that included all types of Enterobacteriaceae (i.e., CRE compared with CSE). In contrast, evidence from outside the United States shows increased mortality with CRE. A case-control study from Israel found that, after adjusting for severity of illness, patients with infections from carbapenem-resistant *K. pneumoniae* had 4 times the risk of death as patients infected with carbapenem-susceptible *K. pneumoniae* [29].

While it seems intuitive that CRE would be associated with worse outcomes, the lack of consistency across the literature raises several important questions about how CRE cases are defined and how CRE outcomes are measured. First, only 2 studies in this systematic literature review restricted cases to patients with CRE-positive cultures from normally sterile body sites. As a result, there is potential that patients colonized with CRE (i.e., a positive culture from a non-sterile site in the absence of signs or symptoms of infection) were included as “infected” cases. Such a misclassification bias could obscure differences in outcomes. As with any bacteria, the body site of infection is a key determinant of outcomes. A CRE bacteremia, for example, would be expected to have a higher mortality rate than a CRE urinary tract infection. In fact, a case-control study from Israel found that carbapenem-resistant *K. pneumoniae* bacteremia has an attributable mortality of 50%, or 3 times the risk of death compared with non-bacteremic controls [30]. Second, patients with CRE may have a higher risk of death for reasons beyond the infection itself. For example, CRE-positive patients may have a higher burden of comorbidities and, due to these comorbidities, be more acutely ill when they do become infected. These factors were not adequately accounted for in all studies. Third, it is possible that outcomes other than mortality are worse in patients with CRE, but these alternate outcomes have gone unmeasured. For example, none of the studies measured hospital re-admissions and costs.

Another potential explanation for the conflicting mortality results is that the current studies were underpowered to find statistically significant results. Each of the 4 studies that evaluated mortality included fewer than 100 CRE-infected patients. In the future, larger well-designed studies should be performed to assess the association between CRE infection and mortality.

All the studies included in this systematic literature review evaluated outcomes before the Food and Drug Administration’s (FDA) approval of ceftazidime-avibactam and meropenem-vaborbactam. Now that these new and potentially more efficacious agents are available, a re-evaluation of CRE-related outcomes is warranted.

There are several limitations to our meta-analysis. First, definitions of CRE varied across studies. This discrepancy reflects differences in how each study chose to define a CRE case; it also reflects changes in the CDC’s definition for CRE and the CLSI’s breakpoints for carbapenem-resistance. The original CDC definition for CRE did not include ertapenem, which limited its sensitivity for detecting OXA-48-producing CRE. Furthermore, studies that used the pre-2010 CLSI guidelines to define carbapenem-resistance or evaluated just certain species of Enterobacteriaceae may have under-estimated the true incidence of CRE [19, 31]. Thus, we were unable to pool data using meta-analytic techniques. Second, while we excluded studies that used surveillance cultures, clinical cultures reflecting colonization of a non-sterile site were not consistently distinguished from true infections. This may have resulted in an overestimation of the incidence of infection and an underestimation of the risk of mortality. Third, our incidence rates were derived from a limited number of geographic regions and not the entire US. However, 2 studies included multiple states, which should increase the generalizability of our findings. Fourth, outcome studies were limited in both number and quality. For example, 3 of the 4 studies involved ≤2 hospitals, and only 1 adjusted for severity of illness, a key determinant of mortality.

## Conclusions

In conclusion, while the incidence of CRE infections in the United States remains low on a national level, the incidence is highest in LTACs. Several studies have measured the incidence of CRE, but the percentage of CRE that results from carbapenemase-production needs to be better defined. Studies assessing outcomes in CRE-infected patients have been small and have reached conflicting results. Future research should measure a variety of clinical outcomes, should be adequately powered and should adequately adjust for confounders to better assess the full burden of CRE. Specifically, outcomes of CRE infections treated with recently FDA-approved antibiotics warrant further evaluation.

Without adequate studies measuring the burden of CRE infections, proper distribution of resources for research and prevention efforts will be impossible, thereby leaving patients vulnerable to this important emerging pathogen.

## Additional file

Additional file 1: Search Methods. (DOCX 32 kb)

## Abbreviations

CDC: Centers for Disease Control and Prevention
CLSI: Clinical and laboratory standards institute
CRE: carbapenem-resistant Enterobacteriaceae
CRKP: carbapenem-resistant *K. pneumoniae*
CSE: carbapenem-susceptible Enterobacteriaceae
KPC: *Klebsiella pneumoniae* carbapenemase
LOS: length of stay
LTCF: long-term care facility
MesH: Medical Subject Headings
MHT: Modified-Hodge Test
MIC: minimum inhibitory concentration
NHSN: National healthcare safety network
