# Supplementary material for: A systematic review of the epidemiology of carbapenem-resistant Enterobacteriaceae in the United States
Source: Antimicrob Resist Infect Control. 2018 Apr 24;7:55. doi: 10.1186/s13756-018-0346-9 (PMC5926528; doi:10.1186/s13756-018-0346-9)
Supplement: Supplementary file 1 — Search Methods. (DOCX 32 kb) [file 13756_2018_346_MOESM1_ESM.docx]

**Additional file 1: Search Methods**

**Methods:** An experienced health sciences librarian conducted systematic searches in MEDLINE via Ovid, Cochrane Library Databases via Wiley, CINAHL via EBSCO, Scopus, and Web of Science to identify papers published from the inception of the databases to October 2014. The searches were rerun in early February of 2016 to identify newer studies. The librarian used terms associated with carbapenem resistant enterobacteriaceae; created a filter for incidence, prevalence, and mortality using a pre-established incidence/prevalence search filter ([http://www.systematicreviewsjournal.com/content/2/1/68](https://email.uiowa.edu/owa/redir.aspx?C=mldckXvug0KXve1OS0jGKDpYVjWx3tAIRWfFcUFDex6IbHK850VO5OHcp45toO0ro7LkzkjwNpU.&URL=http%3a%2f%2fwww.systematicreviewsjournal.com%2fcontent%2f2%2f1%2f68)); and used a pre-established filter to identify economic studies (<http://www.cadth.ca/media/pdf/H0490_Search_Filters_for_Economic_Evaluations_mg_e.pdf>).

Full search strategies are included in the appendix of this article.

**Adverse Effects/Incidence/Prevalence Filtered Strategies**

**Ovid MEDLINE(R) In-Process & Other Non-Indexed Citations and Ovid MEDLINE(R) 1946 to Present** (Run on September 4, 2014, 6597 results found. Rerun on January 25, 2016, limited to 2014-current, 1693 results.)

1. (Carbapenem resistant enterobacter* or CRE).tw.
2. (Klebsiella pneumoniae carbapenemase* or KPC carbapenemase* or Metallo-beta-lactamase* or Serine carbapenemase* or Oxacillinase*).tw.
3. 1 or 2
4. exp Carbapenems/
5. (Carbapenems or Thienamycins or Imipenem or Meropenem or Doripenem or Ertapenem or (Panipenem and betamipron) or Biapenem).tw.
6. 4 or 5
7. exp Drug Resistance, Bacterial/
8. (Resistan* or nonsusceptib*).tw.
9. 7 or 8
10. 6 and 9
11. (Klebsiella or eaggec or alkalescens-dispar group* or e coli or escherichia or Salmonella or Proteus or Pseudomonas or Chryseomonas or flavimonas or enteric bacteria or paracolobactrum or coliform bacilli or Enterobacter*).tw.
12. exp Pseudomonas/ or exp Pseudomonas Infections/ or exp Enterobacteriaceae Infections/ or exp Enterobacteriaceae/
13. 11 or 12
14. 10 and 13
15. 3 or 14
16. exp morbidity/ or exp mortality/
17. exp Epidemiology/
18. exp Epidemiologic Studies/
19. (ae or co or de or ct).fs.
20. (adverse adj2 (effect or effects or reaction or reactions or event or events or outcome or outcomes)).tw.
21. (morbidit* or mortalit* or incidence or prevalence or "length of stay" or readmission or readmit* or "cause of death" or "fatal outome*" or survival or safe or safety or "side effect*" or "undesirable effect*" or "treatment emergent" or tolerability or toxicity or adrs).tw.
22. (occur* or frequenc* or proportion* or rate* or number* or percent*).ti,ab.
23. exp "Length of Stay"/
24. exp Patient Readmission/
25. or/16-24
26. 15 and 25
27. limit 26 to animals
28. limit 26 to humans
29. 27 and 28
30. 27 not 29
31. 26 not 30
32. limit 31 to yr="2000 -Current"

**CINAHL via EBSCO** (Run on September 16, 2014, 679 results. Rerun on January 25, 2016, limited to 2014-2016, 235 results.)

1. MH "Morbidity+" OR MH "Mortality+" OR MH "Epidemiological Research+" OR MH "Epidemiology+" OR MH "Length of Stay" OR MH "Readmission"
2. MW “ae” OR MW “co” OR MW “ct” OR MW “de”
3. adverse N2 (effect OR effects OR reaction OR reactions OR event OR events OR outcome OR outcomes)
4. morbidit* OR mortalit* OR incidence OR prevalence OR “length of stay” OR readmission OR readmit* OR “cause of death” OR “fatal outome*” OR survival OR safe OR safety OR side effect* OR undesirable effect* OR “treatment emergent” OR tolerability OR toxicity OR adrs
5. TI ( (occur* OR frequenc* OR proportion* OR rate* OR number* OR percent*) ) OR AB ( (occur* OR frequenc* OR proportion* OR rate* OR number* OR percent*) )
6. S1 OR S2 OR S3 OR S4 OR S5
7. "Carbapenem resistant enterobacter*" OR CRE
8. “Klebsiella pneumoniae carbapenemase*” OR “KPC carbapenemase*” or “Metallo-beta-lactamase*” OR “Serine carbapenemase*” OR Oxacillinase*
9. S7 OR S8
10. MH "Carbapenems+"
11. Carbapenems OR Thienamycins or Imipenem or Meropenem or Doripenem or Ertapenem or (Panipenem and betamipron) or Biapenem
12. S10 OR S11
13. MH "Drug Resistance, Microbial+"
14. Resistan* or nonsusceptib*
15. S13 OR S14
16. MH "Enterobacteriaceae+" OR MH "Enterobacteriaceae Infections+" OR (MH "Pseudomonas") OR (MH "Pseudomonas Infections")
17. Klebsiella OR eaggec OR “alkalescens-dispar group*” OR “e coli” OR escherichia OR Salmonella OR Proteus OR Pseudomonas OR Chryseomonas OR flavimonas OR “enteric bacteria” OR paracolobactrum OR “coliform bacilli” OR Enterobacter*
18. S16 OR S17
19. S12 AND S15 AND S18
20. S9 OR S19
21. S20 AND S6
22. Limit to 2000-Present

**Cochrane** (Run on September 26, 2014—CDSR 1, DARE 2, CENTRAL 59 results. Rerun on January 29, 2016, CDSR 2, DARE 2, CENTRAL 138 results. )

1. MeSH descriptor: [Morbidity] explode all trees
2. MeSH descriptor: [Mortality] explode all trees
3. MeSH descriptor: [Epidemiology] explode all trees
4. MeSH descriptor: [Epidemiologic Studies] explode all trees
5. MeSH descriptor: [Length of Stay] explode all trees
6. MeSH descriptor: [Patient Readmission] explode all trees
7. adverse near/2 (effect or effects or reaction or reactions or event or events or outcome or outcomes):ti,ab,kw (Word variations have been searched)
8. morbidit* or mortalit* or incidence or prevalence or "length of stay" or readmission or readmit* or "cause of death" or "fatal outome*" or survival or safe or safety or "side effect*" or "undesirable effect*" or "treatment emergent" or tolerability or toxicity or adrs:ti,ab,kw (Word variations have been searched)
9. (occur* or frequenc* or proportion* or rate* or number* or percent*):ti or (occur* or frequenc* or proportion* or rate* or number* or percent*):ab (Word variations have been searched)
10. #1 OR #2 OR #3 OR #4 OR #5 OR #6 OR #7 OR #8 OR #9
11. "Carbapenem resistant enterobacter*" or CRE:ti,ab,kw (Word variations have been searched)
12. "Klebsiella pneumoniae carbapenemase*" or "KPC carbapenemase*" or "Metallo-beta-lactamase*" or "Serine carbapenemase*" or Oxacillinase*:ti,ab,kw (Word variations have been searched)
13. #11 OR #12
14. MeSH descriptor: [Carbapenems] explode all trees
15. Carbapenems or Thienamycins or Imipenem or Meropenem or Doripenem or Ertapenem or (Panipenem and betamipron) or Biapenem:ti,ab,kw (Word variations have been searched)
16. #14 OR #15
17. MH "Drug Resistance, Microbial+"
18. Resistan* or nonsusceptib*
19. #17 OR #18
20. MeSH descriptor: [Enterobacteriaceae] explode all trees
21. MeSH descriptor: [Enterobacteriaceae Infections] explode all trees
22. MeSH descriptor: [Pseudomonas] explode all trees
23. MeSH descriptor: [Pseudomonas Infections] explode all trees
24. Klebsiella or eaggec or "alkalescens-dispar group*" or "e coli" or escherichia or Salmonella or Proteus or Pseudomonas or Chryseomonas or flavimonas or "enteric bacteria" or paracolobactrum or "coliform bacilli" or Enterobacter*:ti,ab,kw (Word variations have been searched)
25. #20 OR #21 OR #22 OR #23 OR #24
26. #16 AND #19 AND #25
27. #13 or #26
28. #27 and #10

**Web of Science** (Run on September 26, 2014, 6893 Results. Rerun on January 29, 2016, limited to 2014-2016, 1937 results.)

1. "Carbapenem resistant enterobacter*" or CRE

OR

"Klebsiella pneumoniae carbapenemase*" or "KPC carbapenemase*" or "Metallo-beta-lactamase*" or "Serine carbapenemase*" or Oxacillinase*

1. Carbapenems or Thienamycins or Imipenem or Meropenem or Doripenem or Ertapenem or (Panipenem and betamipron) or Biapenem

AND

Resistan* or nonsusceptib*

AND

Klebsiella or eaggec or "alkalescens-dispar group*" or "e coli" or escherichia or Salmonella or Proteus or Pseudomonas or Chryseomonas or flavimonas or "enteric bacteria" or paracolobactrum or "coliform bacilli" or Enterobacter*

1. 1 OR 2
2. adverse Near/2 (effect or effects or reaction or reactions or event or events or outcome or outcomes)

OR

morbidit* or mortalit* or incidence or prevalence or “length of stay” or readmission or readmit* or “cause of death” or “fatal outome*” OR survival OR safe or safety or “side effect*” or “undesirable effect*” or “treatment emergent” or tolerability or toxicity or adrs

OR

(occur* OR frequenc* OR proportion* OR rate* OR number* OR percent*) – in topic since abstract isn’t an option.

1. 3 AND 4

**Scopus** (Run on October 2, 2014, 11.367 results. Rerun on February 1, 2016, limited to 2014-present, 3054 results.)

1. "Carbapenem resistant enterobacter*" or CRE

OR

"Klebsiella pneumoniae carbapenemase*" or "KPC carbapenemase*" or "Metallo-beta-lactamase*" or "Serine carbapenemase*" or Oxacillinase*

1. Carbapenems or Thienamycins or Imipenem or Meropenem or Doripenem or Ertapenem or (Panipenem and betamipron) or Biapenem

AND

Resistan* or nonsusceptib*

AND

Klebsiella or eaggec or "alkalescens-dispar group*" or "e coli" or escherichia or Salmonella or Proteus or Pseudomonas or Chryseomonas or flavimonas or "enteric bacteria" or paracolobactrum or "coliform bacilli" or Enterobacter*

1. 1 OR 2
2. adverse W/2 (effect or effects or reaction or reactions or event or events or outcome or outcomes)
3. morbidit* or mortalit* or incidence or prevalence or “length of stay” or readmission or readmit* or “cause of death” or “fatal outome*” OR survival OR safe or safety or “side effect*” or “undesirable effect*” or “treatment emergent” or tolerability

OR

toxicity or adrs

1. (occur* OR frequenc* OR proportion* OR rate* OR number* OR percent*) – in title abstract only
2. #4 OR #5 OR #6
3. #3 AND #7

**Economics Filtered Strategies**

**Ovid MEDLINE(R) In-Process & Other Non-Indexed Citations and Ovid MEDLINE(R) 1946 to Present** (Run on September 26, 2014, 176 Results found. Rerun on January 25, 2016, limited to 2014-current, 63 results.)

1. (Carbapenem resistant enterobacter* or CRE).tw.
2. (Klebsiella pneumoniae carbapenemase* or KPC carbapenemase* or Metallo-beta-lactamase* or Serine carbapenemase* or Oxacillinase*).tw.
3. 1 or 2
4. exp Carbapenems/
5. (Carbapenems or Thienamycins or Imipenem or Meropenem or Doripenem or Ertapenem or (Panipenem and betamipron) or Biapenem).tw.
6. 4 or 5
7. exp Drug Resistance, Bacterial/
8. (Resistan* or nonsusceptib*).tw.
9. 7 or 8
10. 6 and 9
11. (Klebsiella or eaggec or alkalescens-dispar group* or e coli or escherichia or Salmonella or Proteus or Pseudomonas or Chryseomonas or flavimonas or enteric bacteria or paracolobactrum or coliform bacilli or Enterobacter*).tw.
12. exp Pseudomonas/ or exp Pseudomonas Infections/ or exp Enterobacteriaceae Infections/ or exp Enterobacteriaceae/
13. 11 or 12
14. 10 and 13
15. 3 or 14
16. economics/
17. exp "costs and cost analysis"/
18. economics, dental/
19. exp "economics, hospital"/
20. economics, medical/
21. economics, nursing/
22. economics, pharmaceutical/
23. (economic$ or cost or costs or costly or costing or price or prices or pricing or pharmacoeconomic$).ti,ab.
24. (expenditure$ not energy).ti,ab.
25. (value adj1 money).ti,ab.
26. budget$.ti,ab.
27. or/16-26
28. ((energy or oxygen) adj cost).ti,ab.
29. (metabolic adj cost).ti,ab.
30. ((energy or oxygen) adj expenditure).ti,ab.
31. or/28-30
32. 27 not 31
33. 15 and 32
34. limit 33 to yr="2000 - 2015"
35. limit 34 to animals
36. limit 34 to humans
37. 35 and 36
38. 35 not 37
39. 34 not 38

**CINAHL Economic** (Run on October 2, 2014, 182 citations found. Rerun on January 25, 2016, limited to 2014-2016, 55 results)

1. MH "Economics+" OR MH "Costs and Cost Analysis+"
2. TI ( Economic* or cost or costs or costly or costing or price or prices or pricing or pharmacoeconomic* ) OR AB ( Economic* or cost or costs or costly or costing or price or prices or pricing or pharmacoeconomic* )
3. TI ( expenditure* not energy ) OR AB ( expenditure* not energy )
4. TI value N1 money OR AB value N1 money
5. TI budget* OR AB budget*
6. S1 OR S2 OR S3 OR S4 OR S5
7. “Energy cost*” OR “oxygen cost*” OR “metabolic cost*” OR “Energy expenditure*” OR “oxygen expenditure*”
8. S6 NOT S7
9. ( "Carbapenem resistant enterobacter*" OR CRE ) OR ( “Klebsiella pneumoniae carbapenemase*” OR “KPC carbapenemase*” or “Metallo-beta-lactamase*” OR “Serine carbapenemase*” OR Oxacillinase* )
10. MH "Carbapenems+"
11. Carbapenems OR Thienamycins or Imipenem or Meropenem or Doripenem or Ertapenem or (Panipenem and betamipron) or Biapenem
12. S10 OR S11
13. MH "Drug Resistance, Microbial+"
14. Resistan* or nonsusceptib*
15. S13 OR S14
16. MH "Enterobacteriaceae+" OR MH "Enterobacteriaceae Infections+" OR (MH "Pseudomonas") OR (MH "Pseudomonas Infections")
17. Klebsiella OR eaggec OR “alkalescens-dispar group*” OR “e coli” OR escherichia OR Salmonella OR Proteus OR Pseudomonas OR Chryseomonas OR flavimonas OR “enteric bacteria” OR paracolobactrum OR “coliform bacilli” OR Enterobacter*
18. S16 OR S17
19. S12 AND S15 AND S18
20. S9 OR S19
21. S8 AND S20
22. S8 AND S20 -Limiters - Published Date: 20000101-20141231

**Cochrane Economics** (Run on September 26, 2014, CDSR 0, DARE 0, CENTRAL 5. Rerun on January 29, 2016, CDSR 1, DARE 0, CENTRAL 9 results.)

1. "Carbapenem resistant enterobacter*" or CRE:ti,ab,kw (Word variations have been searched)
2. "Klebsiella pneumoniae carbapenemase*" or "KPC carbapenemase*" or "Metallo-beta-lactamase*" or "Serine carbapenemase*" or Oxacillinase*:ti,ab,kw (Word variations have been searched)
3. #1 OR #2
4. MeSH descriptor: [Carbapenems] explode all trees
5. Carbapenems or Thienamycins or Imipenem or Meropenem or Doripenem or Ertapenem or (Panipenem and betamipron) or Biapenem:ti,ab,kw (Word variations have been searched)
6. #4 OR #5
7. MH "Drug Resistance, Microbial+"
8. Resistan* or nonsusceptib*
9. #7 OR #8
10. MeSH descriptor: [Enterobacteriaceae] explode all trees
11. MeSH descriptor: [Enterobacteriaceae Infections] explode all trees
12. MeSH descriptor: [Pseudomonas] explode all trees
13. MeSH descriptor: [Pseudomonas Infections] explode all trees
14. Klebsiella or eaggec or "alkalescens-dispar group*" or "e coli" or escherichia or Salmonella or Proteus or Pseudomonas or Chryseomonas or flavimonas or "enteric bacteria" or paracolobactrum or "coliform bacilli" or Enterobacter*:ti,ab,kw (Word variations have been searched)
15. #10 OR #11 OR #12 OR #13 OR #14
16. #6 AND #9 AND #15
17. #3 OR #16
18. MeSH descriptor: [Economics] explode all trees
19. MeSH descriptor: [Costs and Cost Analysis] explode all trees
20. Economic* or cost or costs or costly or costing or price or prices or pricing or pharmacoeconomic* OR ( expenditure* not energy)
21. value near/2 money
22. budget*
23. #18 OR #19 OR #20 OR #21 OR #22
24. “Energy cost*” OR “oxygen cost*” OR “metabolic cost*” OR “Energy expenditure*” OR “oxygen expenditure*”
25. #23 NOT #24
26. #17 AND #25

**NHS EED Economics** (Run on September 26, 2014, 1 result found. Rerun on January 29, 2016, 1 result.)

1. "Carbapenem resistant enterobacter*" or CRE:ti,ab,kw (Word variations have been searched)
2. "Klebsiella pneumoniae carbapenemase*" or "KPC carbapenemase*" or "Metallo-beta-lactamase*" or "Serine carbapenemase*" or Oxacillinase*:ti,ab,kw (Word variations have been searched)
3. #1 OR #2
4. MeSH descriptor: [Carbapenems] explode all trees
5. Carbapenems or Thienamycins or Imipenem or Meropenem or Doripenem or Ertapenem or (Panipenem and betamipron) or Biapenem:ti,ab,kw (Word variations have been searched)
6. #4 OR #5
7. MH "Drug Resistance, Microbial+"
8. Resistan* or nonsusceptib*
9. #7 OR #8
10. MeSH descriptor: [Enterobacteriaceae] explode all trees
11. MeSH descriptor: [Enterobacteriaceae Infections] explode all trees
12. MeSH descriptor: [Pseudomonas] explode all trees
13. MeSH descriptor: [Pseudomonas Infections] explode all trees
14. Klebsiella or eaggec or "alkalescens-dispar group*" or "e coli" or escherichia or Salmonella or Proteus or Pseudomonas or Chryseomonas or flavimonas or "enteric bacteria" or paracolobactrum or "coliform bacilli" or Enterobacter*:ti,ab,kw (Word variations have been searched)
15. #10 OR #11 OR #12 OR #13 OR #14
16. #6 AND #9 AND #15
17. #3 OR #16

**Web of Science Economics** (Run on October 3, 2014 – 287 Results. Rerun on January 29, 2016, limited to 2014-2016, 91 results)

1. TS=(Economic* or cost or costs or costly or costing or price or prices or pricing or pharmacoeconomic* OR (expenditure* not energy) OR (value Near/2 money) OR budget*) NOT TS=(“Energy cost*” OR “oxygen cost*” OR “metabolic cost*” OR “Energy expenditure*” OR “oxygen expenditure*”)
2. "Carbapenem resistant enterobacter*" or CRE

OR

"Klebsiella pneumoniae carbapenemase*" or "KPC carbapenemase*" or "Metallo-beta-lactamase*" or "Serine carbapenemase*" or Oxacillinase*

1. Carbapenems or Thienamycins or Imipenem or Meropenem or Doripenem or Ertapenem or (Panipenem and betamipron) or Biapenem

AND

Resistan* or nonsusceptib*

AND

Klebsiella or eaggec or "alkalescens-dispar group*" or "e coli" or escherichia or Salmonella or Proteus or Pseudomonas or Chryseomonas or flavimonas or "enteric bacteria" or paracolobactrum or "coliform bacilli" or Enterobacter*

1. 2 OR 3
2. 1 AND 4

**Scopus Economics Search** (Run on October 3, 2014, 715 results found. Rerun on February 1, 2016, Limited to 2014 – present, 95 results.)

1. "Carbapenem resistant enterobacter*" or CRE

OR

"Klebsiella pneumoniae carbapenemase*" or "KPC carbapenemase*" or "Metallo-beta-lactamase*" or "Serine carbapenemase*" or Oxacillinase*

1. Carbapenems or Thienamycins or Imipenem or Meropenem or Doripenem or Ertapenem or (Panipenem and betamipron) or Biapenem

AND

Resistan* or nonsusceptib*

AND

Klebsiella or eaggec or "alkalescens-dispar group*" or "e coli" or escherichia or Salmonella or Proteus or Pseudomonas or Chryseomonas or flavimonas or "enteric bacteria" or paracolobactrum or "coliform bacilli" or Enterobacter*

1. 1 OR 2
2. Economic* or cost or costs or costly or costing or price or prices or pricing or pharmacoeconomic* OR budget* OR (expenditure* not energy) OR (value W/2 money)

NOT

Energy cost*” OR “oxygen cost*” OR “metabolic cost*” OR “Energy expenditure*” OR “oxygen expenditure*”

1. #3 and #4
